# Differential Phosphoproteome Regulation of Nucleus Accumbens in Environmentally Enriched and Isolated Rats in Response to Acute Stress

**DOI:** 10.1371/journal.pone.0079893

**Published:** 2013-11-22

**Authors:** Xiuzhen Fan, Dingge Li, Yafang Zhang, Thomas A. Green

**Affiliations:** Center for Addiction Research, Department of Pharmacology and Toxicology, The University of Texas Medical Branch, Galveston, Texas, United States of America; Max Planck Institute of Psychiatry, Germany

## Abstract

Increasing evidence shows that stress contributes to the pathogenesis of major depressive disorder which is a severe neuropsychiatric disorder and influences over 10% of the world's population. Our previous studies revealed that rats reared in an enriched environment display less depression-related behavior compared to rats raised in an isolated environment, which implies that environmental enrichment produces an antidepressant-like behavioral phenotype. However, the molecular mechanisms are not fully understood. Protein phosphorylation rapidly changes signaling pathway function and alters the function of proteins associated with the stress-induced depressive disorder. Thus, in this study, a phosphoproteomic approach was used to uncover differential phosphoprotein regulation in rat nucleus accumbens between isolated (IC) and enriched environmental conditions (EC) under basal conditions, and in response to acute stress. We found 23 phosphoproteins were regulated in EC vs. IC rats under basal conditions; 10 phosphoproteins regulated by stress in IC rats; and 15 regulated by stress in EC rats. Among all significantly regulated phosphoproteins, 11 of them were represented in at least two conditions. The regulated phosphoproteins represent signaling pathway proteins (including ERK2), enzymes, transcriptional regulators, protein translation regulators, transporters, chaperones and cytoskeletal proteins. These findings provide a global view for further understanding the contribution of protein phosphorylation in depression pathogenesis and antidepressant action.

## Introduction

Increasing evidence shows that stress contributes to the pathogenesis of major depression, which is a severe neuropsychiatric disorder that influences about 10% of the world population [1]. Preclinical and clinical studies indicate that around 20% to 25% of people who experience major stressful events develop major depression, and most severe events rapidly lead to depression in women [2], [3]. The depressed patient is impaired in reward and decision-making [4].

Molecular and cellular studies in rodents indicate that chronic stress leads to a depressive phenotype which is associated with changes in neurochemicals and several proteins in nucleus accumbens (NAc). The findings also reveal NAc plays an important role in stress-induced depression [4]. However, the molecular mechanisms of stress-induced pathological changes contributing to depression remain unknown.

Studies demonstrate that isolated and enriched environmental rearing conditions differentially influence behavior in a multitude of animal models, with enrichment producing protective phenotypes for both addiction- and depression-like behavior [5]–[9]. Specifically, environmental enrichment increases mobility time in the forced swim test modeling a decrease in “behavioral despair”, increases sucrose preference as a model of decreased anhedonia, and increases social grooming as a model of decreased social withdrawal [9]. Additionally, environmental enrichment decreases endoplasmic reticulum stress responses to psychological stress [10], [11]. Environmental enrichment also reverses depression-like behavior in mice deficient in brain-derived neurotrophic factor [12]. It is well known that the NAc has a role in mediating natural reward (and thus anhedonia) [13]–[17], and accumulating evidence also points to a role for NAc in mediating aversive states [18]–[21]. Further, deep brain stimulation of NAc in a clinical study was shown to have antidepressant, antianxiety and antianhedonic effects in depressed patients [22]. Thus, it is of great interest to probe the molecular mechanisms of NAc in response to stressful stimuli in EC vs. IC rats in order to further elucidate the role of NAc in stress-induced depression and antidepressant action.

One might wonder if it is enrichment that produces an antidepressant-like phenotype or does isolation produce a pro-depressant-like phenotype? As always, perspective depends upon one's point of view: if one sees enrichment as the more “natural” condition then one might say that isolation is pro-depressant rather than enrichment being antidepressant. The distinction is purely semantic and readers are encouraged to interpret the data in whichever direction suits their need.

Protein phosphorylation plays an important role in signaling processes and regulation of protein function, which includes subcellular localization, protein degradation/stabilization and biochemical activity [23]. Evidence has shown that signaling pathway proteins are involved in stress-induced depressive disorder in humans and animal models. These include: down-regulated protein kinase A in NAc of young suicide victims [4]; down regulated phosphorylated MAP kinase in rat PFC and hippocampus in response to chronic forced swim stress [24]; and regulation of phosphoCREB level in NAc associated with depression-like behavior in rats [9]. All of this evidence indicates the importance of phosphorylated proteins in the pathogenesis of stress-induced depression. Thus, in the current study, we use a phosphoproteomic approach to uncover changes in phosphorylation in rat NAc by comparing EC vs. IC basal differences and each after acute stress. This study opens a new avenue for further understanding the molecular mechanisms of protein phosphorylation in stress-induced depression pathogenesis and antidepressant action.

## Materials and Methods

### Materials

ProQ diamond phosphoprotein stain was purchased from Invitrogen (Eugene, OR). Precast immobilized DryStrips (pH 3–11NL, 11 cm) and IPG buffer (pH 3–11NL) were purchased from GE Healthcare (Uppsala, Sweden). 2D protein extraction buffer-III was purchased from GE Healthcare (Piscataway, NJ). SDS–Tris–HCI gradient gel (10–20%), Coomassie Blue G250 stain solution and protein solubilization buffer (PSB) were purchased from Bio-Rad (Hercules, CA). Primary antibody for anti-enolase1 was purchased from Cell Signaling (Danvers, MA); anti-phospho-Ser/Thr/Tyr IgG antibody produced from mouse was purchased from AnaSpec (Fremont, CA). HRP linked anti-mouse IgG was purchased from Cell Signaling (Danvers, MA). Chemiluminescent luminal reagent was purchased from GE healthcare (Piscataway, NJ). Creatine kinase activity colorimetric assay kit was purchased from BioVision (Milpitas, CA). The other chemicals used were purchased from Sigma-Aldrich (St. Louis, MO) and were of analytical grade.

### Animal treatment

Twenty four male Sprague-Dawley rats (Harlan laboratories Inc, Houston), 21 days of age, were divided to two conditions (isolated condition and enriched condition), twelve of each. For the IC group, the rats were separated one rat per cage in standard polycarbonate cages with no access to social contact or novelty, whereas EC rats were housed together 12 per cage and with novel toys changed every day. Food and water were freely available for rats and all rats were maintained in a controlled room environment (temperature, 22°C; relative humidity, 50%; and 12 h light/dark cycles) for 40 days prior to acute stress experiment.

Restraint stress: Six rats from each group were placed individually into plastic conical sleeves (Decapi-Cone; model DC200; Braintree Scientific, Braintree, MA) for 30 min. The other six rats in each group remained undisturbed as controls. Stressed rats were then decapitated immediately at the end of stress. The NAc was dissected on an ice-cold platform and stored at −80°C until further analysis [25], [10], [11].

The experiments were performed in accordance with the guidelines of National Institutes of Health and approved by the Institutional Animal Care and Use Committee of The University of Texas Medical Branch at Galveston.

### Protein extraction

The NAc of two rats of the same group was pooled together in one tube for protein extraction due to its limited size. The tissue samples from controls and stress rats of both groups were first washed with ice cold tris buffered saline (TBS) and then homogenized in a buffer [TBS pH 7.4, 1% Igepal-CA630 (NP-40), 1X protease inhibitor cocktail, 20 mM NaF, 1 mM Na3VO4, 10 mM DTT and 5 mM EDTA] on ice, and then the homogenates were centrifuged at 750× g for 20 min at 4°C to remove cell debris. The top fraction was transferred to a new tube and centrifuged at 20,000× g for 20 min at 4°C, the resulting supernatant was taken out and mixed with 1% streptomycin sulfate to remove DNA contaminants [26]. The supernatant, after cleaning up DNA contamination, was then added four volumes of methanol and 1 volume of chloroform (Vprotein:Vmethanol:Vchloroform  = 1∶4∶1) and was incubated at room temperature 15 to 30 min. (vortexing every 5 min) to remove lipids. The sample was centrifuged at 16000× g for 20 min at 4°C. The pellet was then washed with 3% HCI/Acetone to remove the methanol and chloroform (cytosol fraction; [27]–[29]; the procedure for delipidation of the 20,000× g pellet fraction was the same as the supernatant, which produced a crude membrane fraction. Both fractions were then dissolved in a buffer containing 20 mM Tris–HCl pH 7.4, 6 M urea, 1% NP-40, 1× protease inhibitor cocktail, 20 mM NaF, 1 mM Na3VO4, 10 mM DTT and 5 mM EDTA. The protein extracts of both fractions were then subjected to further proteomic analysis.

### 2D gel electrophoresis and ProQ Diamond phosphoprotein stain

Four hundred micrograms of cytosolic protein from control and stress rats of both groups were dissolved in 200 μl PSB and mixed with 100 mM DTT, a trace amount of bromophenol blue and 1% IPG buffer, pH 3–11 NL, and incubated at 21°C for 1 hr. Three hundred fifty micrograms of protein from the membrane fraction were dissolved in 200 μl of 2D protein extraction buffer-III and mixed with 100 mM DTT, trace amount of bromophenol blue and 1% IPG buffer, pH 3–11 NL, and incubated at 21°C for 1 hr. The proteins were then rehydrated to the DryStrip (11 cm, pH 3–11 NL) overnight at the same temperature. For the first dimension, isoelectric focusing (IEF) was performed at 20°C using an Ettan IPGphor3 (GE Healthcare, Sweden) in the following steps: 200 V for 30 min., 500 V for 1.5 hrs, 1000 V for 1.5 hrs, 8000 V for 2.0 hrs and 8000 V for 24000 Vhr. The strips were then equilibrated for 1 hr in equilibration buffer (50 mM Tris–HCl pH 8.8, 6 M urea, 20 mM iodoacetamide, 2% SDS and 20% glycerol). After rinsing two times with SDS–PAGE running buffer, the strips were loaded onto 10–20% SDS-Tris-glycine gradient gels (13.3×8.7×1 cm) and were then subjected to 150 V for 2 hrs and 20 min at room temperature for the 2nd dimension separation. Following the electrophoresis, the gel of each sample (N = 3 samples per group [*i.e*. 6 rats]) was subjected to sequential steps: first, the gel was fixed with 100 ml 40% methanol and 10% acetic acid for 30 min. X 2; 100 ml ultrapure water wash 10 min. X 3; then 80 ml ProQ Diamond phosphoprotein stain incubated for 2 hrs; 100 ml 20% acetonitrile, 50 mM sodium acetate pH 4 destained for 30 to 60 min. X 3; and finally 100 ml ultrapure water wash 10 min. X 3. All steps were performed at room temperature. The gels were then scanned.

### Progenesis SameSpots analysis of protein phosphorylation differences

2D gel images of phosphorylated proteins were acquired by Typhoon Trio imaging system (GE Healthcare). The scanning resolution was 50 μm with excitation λ 532 nm and emission λ 580 nm. The images were then analyzed by the software Progenesis SameSpots (Nonlinear Dynamics, version 4.0) which has been judged to be much improved in reproducibility compared to previous generations of 2D gel analysis software [30]. To align the gel images, one of the six gels was chosen as a reference gel. With the help of manually drawn and automated vectors comparing each image to the reference, images were aligned at the pixel level. The program then performed automatic spot detection and background subtraction. The software assigns the same spot on every gel in the analysis identical shape (spot outline) and spot number. Spot volumes were normalized to those of the reference gel to obtain normalized volumes that are comparable across gels. The fold changes of protein phosphorylation between controls and stress rats in IC and EC groups were also determined as described below in the statistical analysis.

### Trypsin digestion, MALDI TOF-TOF MS and Nano-LC MS-MS analysis

To better visualize the spots for excision, the gels were then stained with CBB G250. The differentially phosphorylated spots in controls and stress IC/EC groups were manually excised from the 2D gel. The protein was then digested with trypsin (0.1 μg per spot, Promega) in 10 μl of 25 mM ammonium bicarbonate, pH 8.0, for 6 hrs at 37°C. One μl of digested sample solution was used for MALDI TOF/TOF MS. The data were collected using an Applied Biosystems 5800 MALDI TOF/TOF proteomics analyzer. The instrument was operated in a positive ion reflection mode with mass range from 850 to 3000 Da. The focus mass was set at 1700 Da. For MS data, 2000–4000 laser shots were acquired and averaged from each sample spot. Automatic external calibration was performed using a peptide mixture with reference masses 904.468, 1296.685, 1570.677, and 2465.199. Following MALDI MS analysis, MALDI MS/MS was performed on several (5∼10) abundant ions from each sample spot. A 1 kV positive ion MS/MS method was used to acquire data under post-source decay (PSD) conditions. The instrument precursor selection window was ±3 Da. For MS/MS data, 2000 laser shots were acquired and averaged from each sample spot. Automatic external calibration was performed using reference fragment masses 175.120, 480.257, 684.347, 1056.475, and 1441.635 (from precursor mass 1570.700).

For spots not easily identified via MALDI TOF/TOF MS, a Nano-LC MS/MS (Thermo Finnigan LTQ Orbitrap Velos) technique was used for protein analysis (noted in Tables 1 and 2). Nine μl of digested sample was injected onto a nano trap column (100 um i.d. ×1 cm, C18 PepMap 100) and then into a C18 reverse-phase column (LC Packings, Acclaim PepMap 100 C18, 3 μm) for peptide fragment separation. The flow rate was 400 nL/min with 60 min LC gradient, where mobile phase A was 5% acetonitrile and 0.1% formic acid, and B was 100% acetonitrile with 0.1% formic acid. Eluate from the c18 column was sprayed through a charged emitter tip (PicoTip Emitter, New Objective, 10+/−1 μm) into the mass spectrometer. The parameters for mass data collection were as following: tip voltage at +2.0 kV, FTMS mode for MS acquisition of precursor ions (60,000 resolution setting); and ITMS mode for subsequent MS/MS of top 6 precursors selected.

**Table 1:** Phosphorylation significantly changed proteins of cytosolic fraction of Rats NAc comparing EC vs. IC, EC stress vs. EC, and IC stress vs. IC.

Spot no.	Protein name	Accession no. (NCBI)	Coding gene	Score	Matched peptides no.	Coverage (%)	Phosphorylated Sites Record\predicted *			Variation of Protein phosphorylation	
							Ser	Thr	Tyr	*p*-value	Fold change
***EC vs. IC***											
1	70-Kda Heat Shock Cognate Protein	gi|178847300	HSPA8	133	11	19	0\22	0\13	0\6	0.005	9.3
2	60 kDa heat shock protein, mitochondrial	gi:129378	HSPD1	547	14	21.6	0\15	0\7	0\5	0.024	3.2
3	dihydropyrimidinase-related protein 2	gi|40254595	DPYSL2	100	6	12	2\20	1\7	0\6	0.030	3.7
4	creatine kinase-B	gi|203476	CKB	339	8	28	0\8	0\7	0\3	0.036	3.9
5	alpha-enolase isoform 1	gi|158186649	ENO1	279	9	32	0\8	0\1	0\6	0.061	2.9
***EC stress vs. EC***											
6	alpha-enolase isoform 1	gi|158186649	ENO1	364	8	28	0\8	0\1	0\6	0.002	2.4
7	heat shock 70 kDa protein; Provisional	gi|149041392	HSPA8	231	8	18	0\22	0\13	0\6	0.015	2.4
8	Heat shock cognate 71 kDa protein	gi:123647	HSPA8	453	11	15.1	0\23	0\13	0\6	0.017	1.3
9	creatine kinase-B	gi|203476	CKB	63	5	18	0\8	0\7	0\3	0.018	2.6
10	Succinyl-CoA ligase [ADP-forming] subunit beta, mitochondrial	gi:52788305	SUCLA2	264	8	15.7	1\9	0\3	0\5	0.045	2.1
11	Keratin, type I cytoskeletal 10	gi:116242600	KRT10	649	8	13.1	3\48	0\3	0\7	0.048	1.6
***IC stress vs. IC***											
12	aconitate hydratase, mitochondrial precursor	gi|40538860	ACO2	108	8	13	0\14	0\9	0\7	0.006	1.9
13	ATP synthase subunit alpha, mitochondrial	gi:83300587	ATP5A1	197	5	10.6	0\9	0\6	0\2	0.016	3.1
14	T-complex protein 1 subunit alpha	gi:135536	TCP1	165	7	13	2\12	0\5	0\2	0.017	4.8
15	Eno1 protein	gi|38649320	ENO1	121	6	20	0\8	0\1	0\6	0.033	1.8
16	creatine kinase-B	gi|203476	CKB	190	5	18	0\8	0\7	0\3	0.041	3.9
17	Glyceraldehyde-3-phosphate dehydrogenase	gi:122065190	GAPDH	594	5	14.4	0\7	0\4	0\5	0.042	2.0
18	enolase 3	gi|109468300	ENO3	262	8	27	0\8	0\1	0\6	0.044	3.2

* The record of protein phosphorylated site was from published reference using identified protein name (swiss-prot database), predicted phosphorylated site was acquired using Netphos 2.0 server.

**Table 2:** Phosphorylation significantly changed proteins of crude membrane fraction of Rats NAc in EC vs. IC, EC stress vs. EC, and IC stress vs. IC.

Spot no.	Protein name	Accession no. (NCBI)	Coding gene	MOWSE Score	Matched peptides no.	Cove-rage (%)	Phosphorylated Sites Record\predicted*			Variation of Protein phosphorylation	
							Ser	Thr	Tyr	*p*-value	Fold change
***EC vs. IC***											
19	aspartate aminotransfer-ase	gi|6980972	GOT2	203	11	26	0\14	0\3	0\3	1.43e–004	−3.2
20	cyclic nucleotide phosphodiesterase 1	gi|149054231	CNP	216	7	19	0\14	0\6	0\4	0.003	−2.6
21	RNA-binding protein isoform G3BP-2a	gi|149033807	G3BP2	108	5	14	0\18	0\9	0\5	0.003	−3.1
22	NADH dehydrogenase 1 alpha subcomplex subunit 9, mitochondrial	gi|60688426	NDUFA9	69	5	15	0\17	0\6	0\1	0.010	−2.2
23	actin, cytoplasmic 1	gi|4501885	ACTB	174	7	26	0\11	0\7	0\6	0.015	−2.6
24	heat shock protein HSP 90-beta	gi|40556608	HSP90AB1	599	18	24	2\21	0\8	0\11	0.015	−1.9
25	tubulin beta-5	gi7106439	TUBB	628	7	20	0\12	0\9	0\6	0.018	−3.0
	tubulin alpha-4A chain	gi6678467	TUBA4A	423	6	17	0\10	0\3	0\5	0.018	−3.0
26	polyadenylate-binding protein 1	gi19705459	PABPC1	497.9	8	13	0\23	0\7	0\4	0.019	−3.1
27	ATPase family AAA domain-containing protein 3	gi77917538	ATAD3A	321	5	8	0\18	0\11	0\3	0.023	−3.0
28	aldehyde dehydrogenase, mitochondrial	gi|25990263	ALDH2	219	5	11	0\9	0\3	0\6	0.024	−2.5
29	actin, cytoplasmic 1	gi|4501885	ACTB	542	10	32	0\11	0\7	0\6	0.031	−1.8
30	malate dehydrogenase	gi|15100179	MDH1	187	7	26	0\8	0\6	0\3	0.041	−2.5
31	pyruvate kinase isozymes M1/M2	gi|16757994	PKM1/2	685	19	41	0\15	0\8	0\4	0.042	−1.7
32	pyruvate kinase isozymes M1/M2	gi|16757994	PKM1/2	241	11	26	0\15	0\8	0\4	0.042	−1.5
33	dihydrolipoyllysine-residue succinyltransferase component of 2-oxoglutarate dehydrogenase complex, mitochondrial	gi195927000	DLST	485	7	12	0\11	0\6	0\2	0.001	2.6
	tubulin alpha-1C	gi58865558	TUBA1C	264	3	7	0\8	0\4	0\5	0.001	2.6
34	actin, cytoplasmic 2-like	gi293342999	ACTB	729	11	29	0\11	0\7	0\6	0.006	3.8
35	V-type proton ATPase catalytic subunit A	gi|157819953	ATP6V1A	304	14	26	1\19	0\6	1\9	0.023	1.2
36	tyrosine 3-monooxygenase/tryptophan5-monooxygenase activation protein, epsilon polypeptide	gi|149053421	YWHAE	108	8	44	0\7	0\3	0\5	0.031	3.1
37	unnamed protein product (Albumin)	gi|55628	ALB	144	5	11	0\6	0\10	0\5	0.035	1.5
38	isocitrate dehydrogenase 3 (NAD+) alpha	gi149041704	IDH3A	375	6	17	0\6	0\5	0\6	0.037	1.4
***EC stress vs. EC***											
39	RNA-binding protein isoform G3BP-2a	gi|149033807	G3BP2	156	5	14	0\18	0\9	0\5	0.007	−3.8
40	actin, cytoplasmic 1	gi|4501885	ACTB	376	9	32	0\11	0\7	0\6	0.037	−1.5
41	tubulin beta-2A chain isoform 2	gi|358418568	TUBB2A	85	3	9	0\11	0\6	0\5	0.045	−1.8
42	tubulin beta-2A chain isoform 2	gi|358418568	TUBB2A	241	7	21	0\11	0\6	0\5	0.048	−1.4
43	actin, cytoplasmic 1	gi|4501885	ACTB	174	7	26	0\11	0\7	0\6	0.002	1.8
44	uncharacterized protein LOC364073 (DDX3X)	gi157819755	DDX3X	556	7	11	7\44	0\6	3\12	0.005	1.7
	ATP-dependent RNA helicase DDX3Y	gi264681499	DDX3Y	447	7	11	1\31	1\5	1\14	0.005	1.7
45	V-type proton ATPase catalytic subunit A	gi|157819953	ATP6V1A	304	14	26	1\19	0\6	1\9	0.013	1.2
46	dihydropyrimidinase-related protein 2	gi|40254595	DPYSL2	678	13	35	2\20	1\7	0\6	0.023	2.1
47	dihydropyrimidinase-related protein 2	gi|40254595	DPYSL2	569	12	30	2\20	1\7	0\6	0.035	2.0
48	mitogen-activated protein kinase 1	gi|6754632	MAPK1	72	6	17	0\3	0\3	0\9	0.042	1.5
49	pyruvate kinase isozymes, M1/M2	gi16757994	PKM1/2	956	10	21	0\15	0\8	0\4	0.043	1.9
	synaptotagmin-1	gi148356226	SYT1	462	5	12	0\6	0\6	0\6	0.043	1.9
50	ATPase family AAA domain-containing protein 3	gi77917538	ATAD3A	321	5	8	0\18	0\11	0\3	0.046	2.1
51	pyruvate kinase isozymes M1/M2	gi|16757994	PKM1/2	685	19	41	0\15	0\8	0\4	0.050	2.0
52	unnamed protein product (Enolase 1)	gi|56107	ENO1	535	12	45	0\8	0\2	0\6	0.052	1.2
***IC stress vs. IC***											
53	keratin, type I cytoskeletal 10	gi57012436	KRT10	295.6	5	9	2\31	0\6	0\7	0.014	1.7
54	Gln synthetase	gi|228136	GLNS	201	7	16	0\7	0\1	0\6	0.019	2.1
55	Transcriptional activator protein Pur-alpha	gi|229891503	PURA	242	6	60	3\1	0\0	1\3	0.052	2.3

* The record of protein phosphorylated site was from published reference using identified protein name (swiss-prot database), predicted phosphorylated site was acquired using Netphos 2.0 server.

### Protein identification

Applied Biosystems GPS ExplorerTM (version 3.6) software was employed for searching the respective protein database (NCBI or SwissProtein database) using both MALDI MS and MS/MS spectral data for protein identification. Protein match probabilities were determined by using MASCOT scores. A MASCOT score of more than 64 was considered significant (*i.e*. *p*<0.05). MS peak filtering included the following parameters: mass range 800 Da to 4000 Da, minimum S/N filter  = 10, mass exclusion list tolerance  = 0.5 Da, and mass exclusion list (for some trypsin and keratin-containing compounds) included masses 842.51, 870.45, 1045.56, 1179.60, 1277.71, 1475.79, and 2211.1. For MS/MS peak filtering, the minimum S/N filter  = 10. The mass data were matched to the NCBI protein database. Precursor tolerance was set at 0.2 Da; MS/MS fragment tolerance was set at 0.3 Da; mass  =  monoisotopic; and peptide charges were only considered as +1.

For Nano-LC MS/MS, XCalibur raw data was imported into Thermo Proteome Discoverer1.2.0.208 to search the respective protein database (NCBI or SwissProtein database) using MS/MS spectral data for protein identification. Precursor ion mass tolerance is set at 0.01 Da; MS/MS fragment mass tolerance is set at 0.3 Da; and peptide charges are considered as +2 and +3. The significance of a protein match is accompanied by an expectation value which is the number of matches with equal or better scores that are expected to occur by chance alone. The default significance threshold is p<0.05, so an expectation value of 0.05 is considered to be threshold. However, we used a more stringent threshold of 10^−3^ for protein identification.

### Creatine Kinase Activity colorimetric assay

The creatine kinase B (CKB) activity of the cytosolic fraction was measured with a CK activity colorimetric assay kit which measured the CK forward reaction rate. The dorsal striatum tissue from the same rats was homogenized in a buffer as described in the literature [31] to obtain the cytosolic fraction. Then, Amicon ultra centrifugal filter (filtrates molecular weight <3000 Da) was used to remove small interfering molecules of the extracts, such as ATP, ADP, NADH etc. The assay performance was according to manufacturer's instructions. Briefly, 0.5 μg (per well) cytosolic protein extracts was added to a 96 well flat bottom microplate (Costar, 9018). Samples were run in duplicate. A 50 μl CKB reaction mixture was applied to each well. The CKB converts creatine into phosphocreatine and ADP. The product phosphocreatine and ADP react with the enzyme mixture to form an intermediate that reduces a colorless probe to a colored product with strong absorbance at 450 nm. The absorbance was monitored at 450 nm by a Spectromax M2 microplate reader with a kinetic mode (Molecular Devices).

### Phosphoprotein validation by sandwich ELISA

A flat bottom 96 well microplate (Costar, #9018) was coated with 165 ng (per well) rabbit polyclonal anti-enolase 1 antibody in 50 μl of 0.1 M sodium bicarbonate (pH 9.6) at RT for 3 h. The same antibody was coated in duplex wells. The wells were then washed with 200 μl 0.1 M sodium bicarbonate (pH 9.6) 3X, blocking the nonspecific binding to the well surface with 1% tween20 in TBS pH 7.4 for 2 h at RT, and incubated 20 μg (per well) cytosolic protein extract of the nucleus accumbens, which was the same used in the 2D gel, for 2 h at RT, then washing with 200 μL TBST (TBS pH 7.4+ tween20 0.05%) 3X. Then, 100 μl mouse anti-phospho-Ser/Thr/Tyr antibody (1∶200 diluted) was added to the wells and incubated for 2 h at RT to recognize phospho-enolase 1. The HRP-conjugated horse anti-mouse IgG 100 μl (1∶2000 dilution) was then applied to the wells for 1 h at RT. After washing the wells with 200 μl TBST 5X, 200 μl of chemiluminecent luninol reaction reagent was added to the wells, after incubating for 15 min at RT, the absorbance was then measured at 425 nm with a SpectraMas M2 microplate reader (Molecular Devices).

### Statistical analysis for proteins

The normalized intensity volume of phosphorylated protein spots in 2D gels of basal or stressed EC and IC rats were compared by Student's *t*-test following the Progenesis SameSpots analysis, *p* values <0.05 were considered statistically significant.

CKB activity assay data were analyzed using two-way ANOVAs followed by the post hoc Bonferroni correction. The correlation between CKB activity and normalized intensity of phosphoCKB in 2D gel profiles were analyzed by correlational analysis. The significance was rated at *p*<0.05. The software SPSS was used for the statistical analysis.

### In silico analysis of phosphorylated proteins

The NetPhos2.0 database (http://www.cbs.dt.dk/services/NetPhos/) was used for prediction of protein phosphorylation sites [32]. The phospho-site prediction was the basis of protein sequence searching, with an output score threshold of >0.5. Output scores <0.5 were excluded in order to decrease the probability of false-positives. Next, the significantly-regulated phosphoproteins were analyzed using Ingenuity Pathways Analysis (IPA, http://www.ingenuity.com), which uses the Ingenuity Knowledgebase, setting a cutoff *p* value of <0.05. The IPA analysis yielded significant molecular interaction networks, canonical pathways and biological functions.

## Results

**Figure 1:**
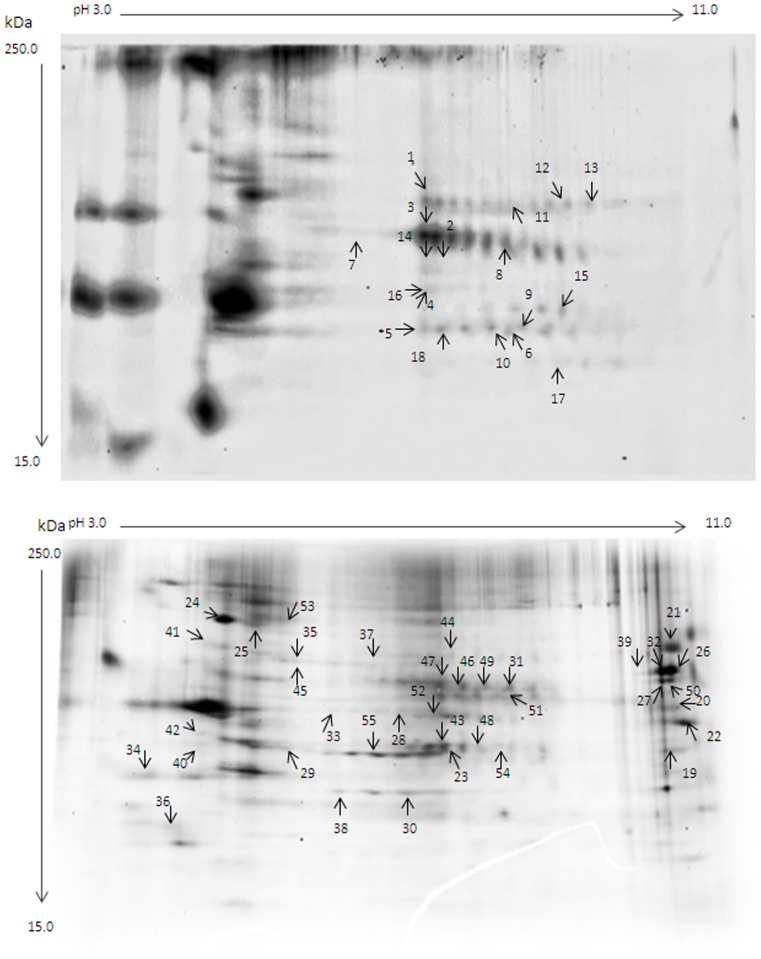
ProQ Diamond stained gels. Representative 2D gels for cytosolic (top panel) and membrane (bottom panel) fractions. Spot numbers correspond to IDs in Tables 1 and 2 respectively.

ProQ diamond fluorescent labeled protein phospho-Ser, phospho-Thr and phospho-Tyr in 2D protein profiles are shown in Fig. 1 (top panel, cytosolic fraction; bottom panel, membrane fraction). The phosphoproteins were then analyzed by Progenesis SameSpots, and a total 55 differentially-phosphorylated protein spots in both fractions were subjected to MALDI TOF/TOF MS/MS and/or Nano-LC MS-MS analysis.

### Altered phosphoproteins in NAc comparing EC vs. IC control rats

A total 23 phosphoproteins were significantly regulated in EC vs. IC control rats, which are shown in Tables 1 and 2 (numbers correspond to Fig. 1, top panel and bottom panel, respectively). Among these, 13 phosphoproteins were decreased in EC rats and 10 phosphoproteins were increased. Decreased proteins included: cytoskeletal proteins ACTB (spot no. 23 and 29), TUBA4A and TUBB (no. 25); enzymes ALDH2 (no. 28), CNP (no. 20), G3BP2 (no. 21), GOT2 (no. 19), MDH1 (no. 30), NDUFA9 (no. 22); signaling pathway proteins ATAD3A (no. 27), PKM2 (no. 31 and 32); the translation regulator PABPC1 (no. 26); and the chaperone protein HSP90AB1 (no. 24). Increased phosphoproteins in EC rats include cytoskeletal proteins actin, cytoplasmic 2 (no. 34), alpha-tubulin 1c (no. 33); signaling pathway proteins YWHAE (no. 36) and CKB (no. 4); enzymes IDH3A (no. 38), DPYSL2 (no. 3), DSLT (no. 33); the H+ transporter ATP6V1A (no. 35 and 45); the glycolysis enzyme ENO1 (no. 5); chaperone proteins, HSPA8 (no. 1) and HSPD1 (no. 2); and the protein albumin (no. 37).

### Altered phosphoproteins in NAc of EC stress vs. EC control rats

In EC rats, stress led to differential phosphorylation of 15 proteins, which are shown in Tables 1 and 2. Interestingly, four upregulated phosphoproteins were unique to EC stress vs. EC control: enzymes DDX3X/Y (no. 44) and SUCLA2 (no. 10); the kinase MAPK1 (a.k.a. ERK2; no. 48); and the vesicular neurotransmitter releasing protein SYT1 (no. 49). Three additional phosphoproteins were regulated by stress in both EC and IC rats: the cytoskeletal protein KRT10 (no. 11); the glycolysis enzyme ENO1 (no. 6 and 52), and the kinase CKB (no. 9); the other 8 phosphoproteins were also found regulated in EC stress vs. EC and also in EC vs. IC control rats. They were: upregulated signaling proteins ATAD3A (no. 27 and 50) and PKM2 (no. 49 and 51); the upregulated H+ transporter ATP6V1A (no. 45 and 35); an upregulated enzyme DPYSL2 (no. 46 and 47); chaperone proteins, the up-regulated HSPA8 (no. 7 and 8) and down-regulated G3BP2 (no. 39); downregulated cytoskeletal proteins, TUBB2A (no. 41 and 42), ACTB (no. 40) and an upregulated ACTB isoform (no. 43).

### Altered phosphoproteins in NAc of IC stress vs. IC control rats

In IC rats, we identified a total of 10 phosphoproteins that were all upregulated by stress in IC rats. Seven of the total 10 phosphoproteins were only regulated in IC stress vs. IC. They were: energy metabolism enzymes ACO2 (no. 12), ENO3 (no. 18), ATP5A1 (no. 13), TCP1 (no. 14), and GAPDH (no. 17); the transcription regulator PURA (no. 55); and the enzyme GLNS (no. 54). Two additional energy metabolism phosphoproteins, ENO1 (no. 15) and CKB (no. 16), and the cytoskeletal protein KRT10 (no. 11 and 36), as mentioned above, were also up-regulated by stress in EC rats.

### Ingenuity Pathways Analysis of regulated phosphoproteins

**Figure 2:**
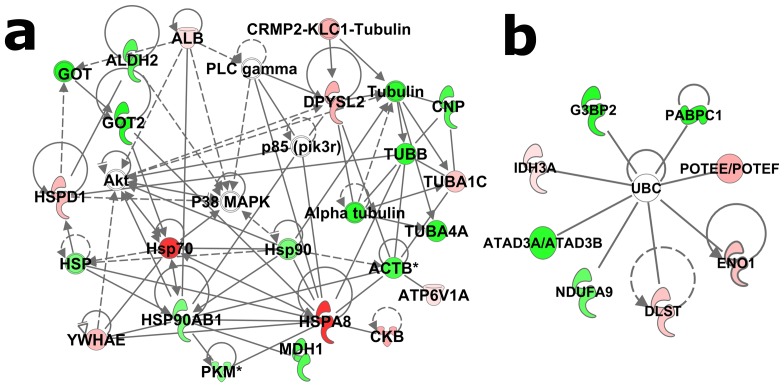
Basal differences in protein phosphorylation between EC and IC rats. The two panels represent separate networks identified by IPA analysis. Red symbols denote elevated phosphorylation in EC rats and green denotes decreased levels of phosphorylation. Intensity of the color represents greater fold change. Asterisks (*) denote multiple regulated spots mapping to the same protein.

When analyzing EC/IC basal differences, 16 of the total 23 phosphoproteins significantly regulated in EC vs. IC rats fit into a network with an IPA network score of 47. This network is dominated by heat-shock proteins and cytoskeletal proteins. Further, this network suggests possible involvement of Akt, p38 MAPK and the p85 (pik3r) kinase signaling pathways (Fig. 2a). In addition to that network, a second network (scoring 18) comprised all of the other seven (of the total 23) phosphoproteins. All of these proteins are targets of ubiquitin C (Fig. 2b). The top-scoring biological functions of the 23 phosphoproteins were associated with nucleic acid metabolism, cellular assembly and cellular organization.

**Figure 3:**
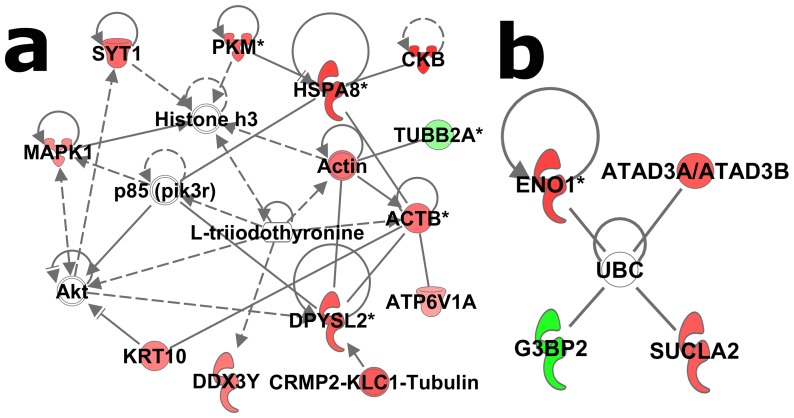
Stress-induced protein phosphorylation in EC rats. The two panels represent separate networks identified by IPA analysis. Red symbols denote elevated phosphorylation in EC stress rats vs. EC control and green denotes decreased levels of phosphorylation. Intensity of the color represents greater fold change. Asterisks (*) denote multiple regulated spots mapping to the same protein.

In EC rats, the most notable change in phosphorylation subsequent to stress was an upregulation in phosphoMAPK1 (pERK2). Additionally, 11 of the total 15 phosphoproteins fit into a network with a score of 31 that suggests possible involvement of Akt, p85 (pik3r) signaling pathway, and histone h3 (Fig. 3a). This network was focused largely on energy production and cytoskeletal organization proteins. Although the Akt and p85 pathways were also identified in the EC/IC basal analysis above, the proteins in these networks were largely non-overlapping: only 4 proteins (DPYSL2, HSPA8, CKB and PKM) were in common for the two networks (Fig 2a and 3a) and only 2 of these proteins (DPYSL2 and HSPA8) have contiguous interactions with Akt or p38. All four phosphoproteins not in the network above are known ubiquitin targets (Fig. 3b; network score of 9). Top biological functions of the 15 phosphoproteins were associated with behavior, cellular assembly, cellular movement, cell death and survival, and connective tissue disorder.

**Figure 4:**
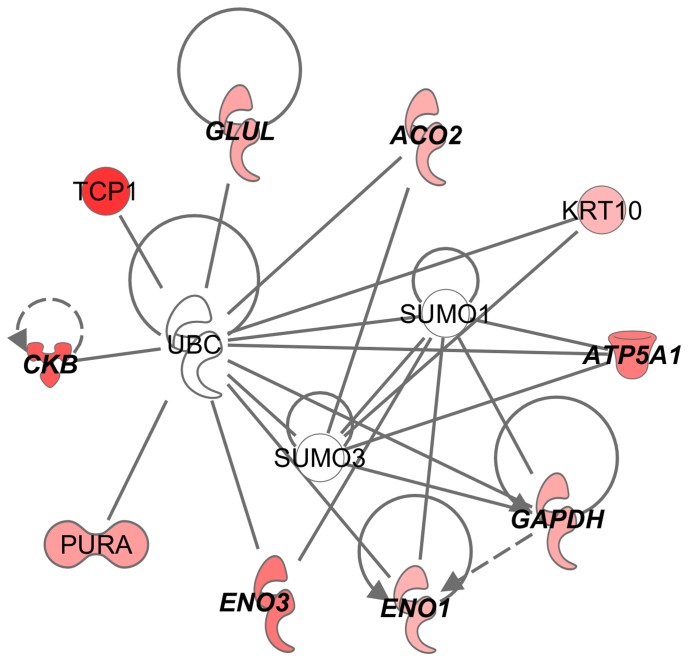
Stress-induced protein phosphorylation in IC rats as identified by IPA. Red symbols denote elevated phosphorylation in IC stress rats vs. IC control and the intensity of the color represents greater fold change. Bold type denotes energy metabolism proteins.

In IC rats, stress led to a total of 10 phosphoproteins upregulated. All of the ten phosphoproteins are targets of ubiquitin C, SUMO1 or SUMO3 (Fig. 4; network score of 28). Top biological functions were associated with nucleic acid metabolism and cell morphology.

**Figure 5:**
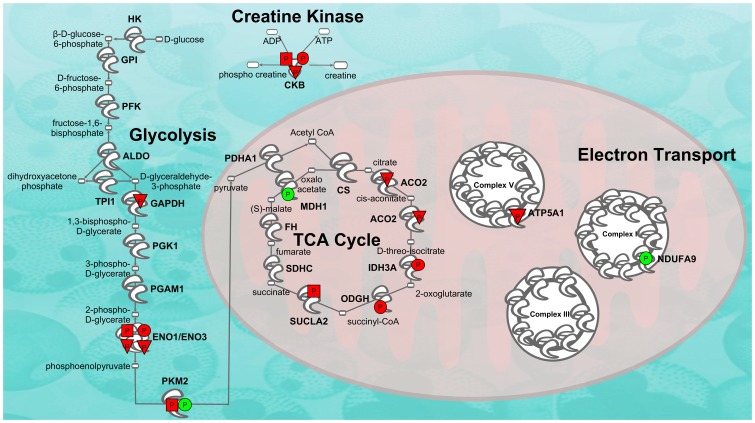
Effect of environmental enrichment and stress on phosphorylation of energy metabolism proteins. Phosphorylation denoted by symbols with “P”. Circles represent EC vs. IC control differences (red denotes higher in EC rats and green lower). Squared symbols represent stress-induced increases in phosphorylation in EC rats and triangles represent stress-induced increases in phosphorylation in IC rats.

A comparison analysis revealed that the only four canonical pathways significantly over-represented in all three comparisons (EC/IC basal, EC stress and IC stress) were related to energy production (glycolysis, gluconeogenesis, tricarboxylic acid cycle and phosphocreatine biosynthesis). Figure 5 depicts differential phosphorylation in these pathways. The results show that stress specifically increases phosphorylation of proteins in these pathways (red triangles and squares).

### Increased CKB activity

**Figure 6:**
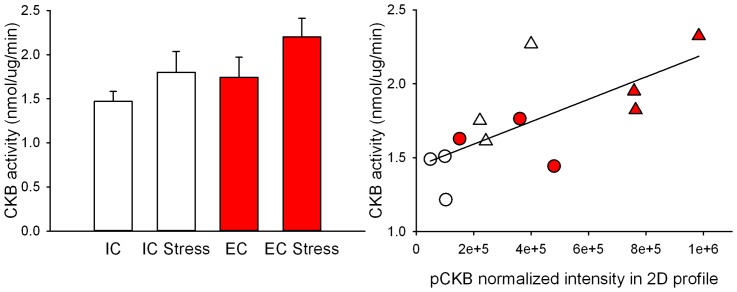
Stress induced CKB activity changes. EC rats had more CKB activity compared to IC rats and stress increases CKB activity. The bar graph (a) represents mean (±SEM) CKB activity for each group, and the scatter plot (b) shows the correlation between normalized intensity of phosphoCKB in 2D gel profile and CKB activity. Triangles represent stress samples and circles represent non-stressed controls. Red symbols represent EC and white symbols IC samples.

Overall, EC rats exhibited increased CKB activity compared to IC rats [main effect of enrichment, F (1, 16)  = 4.803, p<0.05; Figure 6a]. In addition, stress induced CKB activity compared non-stressed rats [main effect of stress, F (1, 16)  = 3.246, p<0.05 (one-tailed test); Fig. 6a]. Thus, stressed rats in the EC condition exhibit the highest CKB activity. The CKB activity of all groups was correlated with normalized intensity of pCKB in the 2D gel profile that was analyzed by a correlational t-test (t = 3.1682, p<0.05, R^2^ = 0.50; Fig. 6b).

### Orthogonal phosphoprotein validation with sandwich ELISA

**Figure 7:**
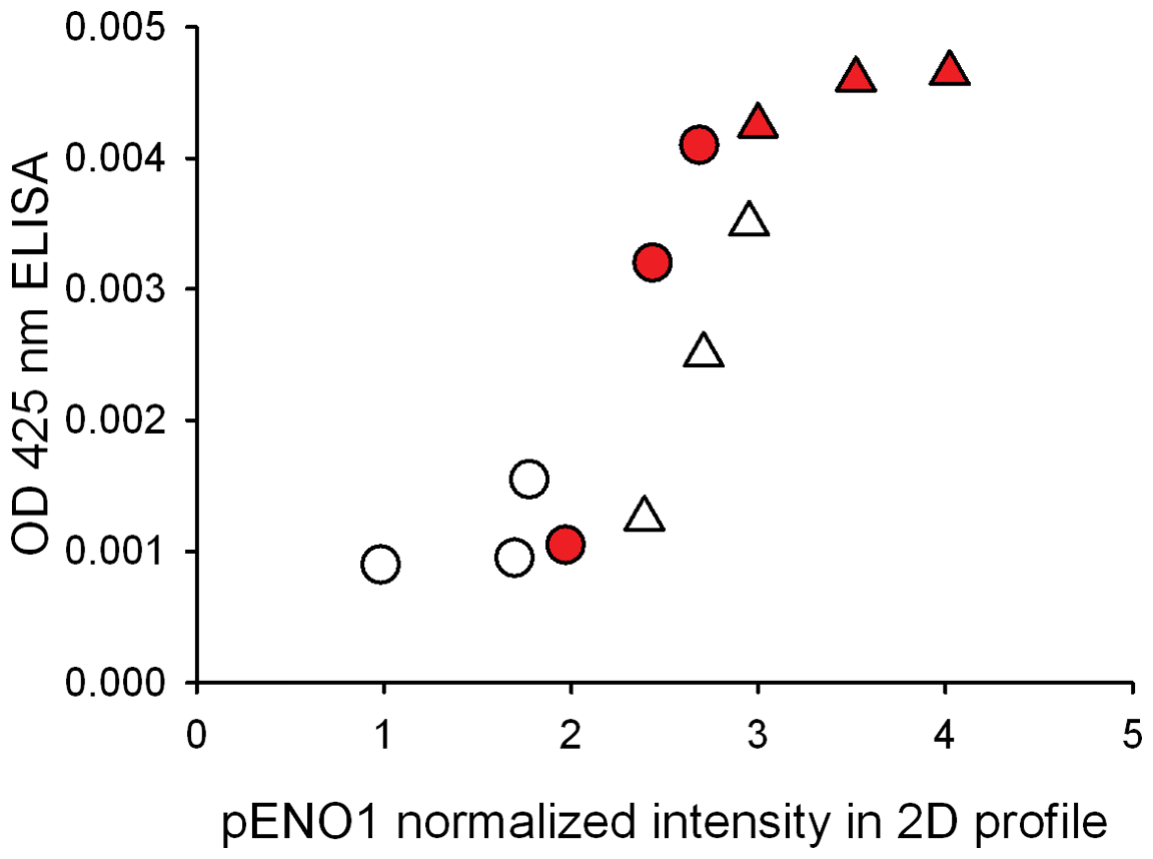
Orthogonal validation of phosphorylation changes. Scatter plot represents correlation in intensity of phosphoENO1 between 2D gels with ProQ Diamond stain (x-axis) and sandwich ELISAs (y-axis). The correlation coefficient was 0.881, *p*<0.01. Triangles represent stress samples and circles represent non-stressed controls. Red symbols represent EC and white symbols IC samples.

To validate our methods and verify differential phosphorylation of a key protein, a sandwich ELISA was performed for pENO1 validation. Results show that phosphorylation of ENO1 from the cytosolic fraction was increased in EC vs. IC control, EC stress vs. EC and IC stress vs. IC, which is consistent with the ProQ diamond 2D gel results (Fig. 7). The correlation coefficient for pENO1 was 0.88, *p*<0.01.

## Discussion

Proteomics has been used broadly in the study of psychiatric and neurological disorders to detect variations in protein expression and post-translational modifications in pathological conditions [33]–[35]. In the current study we employed a two-dimensional gel electrophoresis proteomic technique using the ProQ Diamond stain to resolve differential protein phosphorylation in rat NAc after stress (vs. control) in environmentally enriched and isolated rats. We measured 667 spots total. Interestingly, environmental enrichment led to 23 proteins with differential phosphorylation compared to IC rats under basal conditions. There was a differential response to stress in EC vs. IC rats, meaning that there was little overlap in phosphorylated proteins between EC and IC rats after 30 min stress (Tables 1 and 2). All the regulated phosphoproteins played extensive roles in cellular function, which broadly represent signaling pathway proteins, energy-producing enzymes, transcriptional regulators, a translational regulator, transporters, chaperones and cytoskeletal proteins.

We have previously published a study [36] using these samples investigating differential regulation of total protein (as resolved by Coomassie Brilliant Blue stain). The current study extends upon those results by looking specifically at protein phosphorylation. We found several regulated phosphoproteins were also regulated at the total protein level: HSPA8, HSPD1, DPYSL2, ENO1, SUCLA2, ATP5A1, and GAPDH. If total protein levels are altered, one might wonder if differences in phosphorylation mirror total protein changes. This seems to be the case for 3 of the 4 proteins regulated under basal conditions (HSPA8, HSPD1 and ENO1, but not DPYSL2). SUCLA2, a protein regulated in EC rats after stress was also regulated in the same direction as total protein. However, for ATP5A1, ENO1 and GAPDH in the IC stress comparison, the changes in phosphorylation are opposite to total protein, meaning that phosphorylation increased despite dramatic decreases in total protein amount. It should be noted that all three of these proteins are ubiquitin targets. Although speculative at this point, it is possible that phosphorylation targets these proteins for degradation. This sort of regulation is not without precedent [37]–[39].

Among identified phosphoproteins in this study, ATP5A1, HSPD1, DPYSL2, ENO3, ACO2, CNP, HSP90AB1, ACTBL2, TUBA, TUBB and DDX were also found in dorsolateral prefrontal cortex of major depressive disorder patients [1], which further supports the contention that phosphoproteins contribute to depression pathogenesis. Thus, it will be of great interest to further delineate the roles of these phosphoproteins in depressive disorder in future studies. The molecular interaction network of regulated phosphoproteins demonstrated that stressed EC rats had less of UBC target phosphoproteins than IC rats, and UBC target phosphoproteins were totally different between environmental conditions except for pENO1 (Figure 3, and 4). Moreover, a functional assay for CKB showed that environment enrichment significantly increased CKB activity compared IC rats in either basal or stress condition (Figure 6). The increased CKB activity correlated with increased CKB phosphorylation. The result was consistent with the literature showing that phosphorylation alters CKB activity [40], [41]. Studies reveal that CKB plays an important role in energy homeostasis through the phosphocreatine-creatine kinase system. Enhancement of CKB activity was able to rescue ATP depletion, aggregate formation and impaired proteasome activity in a Huntington's disease mouse model [42]. Thus, it is possible that increased CKB activity in environment enrichment could contribute to the protective depression phenotype of EC rats.

Most research into the role of extracellular signal-regulated kinase (ERK, aka MAPK) in depression has focused on other brain regions [43]–[45]. For example, studies reveal that decreased pERK (pMAPK) in rat hippocampus and prefrontal cortex correlates with depressive-like behavior following chronic forced swim stress [24], and that pERK is down in rat hippocampus of rats after social defeat [46]. There is one report, however, showing that repeated but not acute stress increases pERK2 in the NAc [47]. The current study finds an increase in pERK2 (MAPK1) after acute stress in EC rats but not IC rats.

The pERK2 result described above is consistent with our contention that, even though EC rats are not overcrowded and almost never show aggression toward each other, the EC condition represents a more stressful environment compared to the IC condition. In addition to the pERK2 result, EC rats exhibit lower basal corticosterone levels and blunted corticosterone responses to stressful stimuli [48], [49], both results indicative of chronic mild stress [50], [51]. Further, EC rats show blunted induction of stress-induced endoplasmic reticulum stress genes [11].

Twelve of the regulated proteins (comprising 20 different spots) are involved in energy production, from glycolysis to the citric acid cycle to ATP synthesis (see Figure 5). A rapid change in energy-producing proteins is likely reflective of significant rapid changes in energy demand of the nucleus accumbens. This in turn is very likely a result of changes in neuronal activity because significant amounts of ATP are necessary to return neurons to their normal resting potential after an action potential. Thus, the results of the current studies suggest that EC and IC rats exhibit differential basal amounts of neuronal activity and differential amounts of activity subsequent to stress.

Ubiquitination and the closely related sumoylation post-translational modifications are a recurring theme in the current experiment (see Figures 2b, 3b and 4) as well as our study of total protein levels from these same samples [36], and an ongoing study investigating mRNA from EC and IC rats after cocaine self-administration (in preparation). The evidence suggests that many of the phosphoproteins regulated by stress are ubiquitin target proteins. Current dogma suggests that only about half of the targets of ubiquitination are shuttled to the proteasome, suggesting that non-degradation signaling is involved with the other half [52], [53]. Sumoylation, on the other hand, is thought to be purely a signaling event [53], [54]. At this point it is unclear if many of these regulated proteins are being degraded, but given that phosphorylation is a well-known signaling event, it is likely that ubiquitination and sumoylation are acting as signaling events downstream of the phosphorylation described in these studies.

Individuals vary widely as to their responses to acute stressors. It is thought that these individual differences in responding can determine a person's propensity to develop major depression subsequent to major life stressors. These results confirm and expand upon our previous studies showing that environmental enrichment, a manipulation that produces an antidepressant-like behavioral phenotype in rats [9], also induces individual differences in the rapid phosphoproteomic response of the NAc after an acute stressor. These results provide future avenues for developing therapeutics for the prevention or treatment of major depression.
